# Traditional Herbal Medicine-Derived Sulforaphene LFS-01 Reverses Colitis in Mice by Selectively Altering the Gut Microbiota and Promoting Intestinal Gamma-Delta T Cells

**DOI:** 10.3389/fphar.2017.00959

**Published:** 2018-01-09

**Authors:** Ming Li, Jiali Gao, Yan Tang, Meishuo Liu, Sijin Wu, Kunli Qu, Xiangyu Long, Huajun Li, Min Liu, Yinhui Liu, Jieli Yuan, Lei Mao, Yu Liu, Xiliang Zheng, Erkang Wang, Jin Wang, Yongliang Yang

**Affiliations:** ^1^Department of Microecology, College of Basic Medical Sciences, Dalian Medical University, Dalian, China; ^2^Center for Molecular Medicine, School of Life Sciences and Biotechnology, Dalian University of Technology, Dalian, China; ^3^DrivingForce Therapeutics, Venture Harbor, Dalian, China; ^4^School of Software, Dalian University of Technology, Dalian, China; ^5^State Key Laboratory of Electroanalytical Chemistry, Changchun Institute of Applied Chemistry, Chinese Academy of Sciences, Changchun, China; ^6^Department of Chemistry and Physics, State University of New York, Stony Brook, NY, United States

**Keywords:** colitis, microbiota, sulforaphene, γδT cells, barrier function, herbal medicine

## Abstract

Sulforaphene (LFS-01) is a natural compound derived from traditional herbal medicine. Here, we show that oral administration of LFS-01 is able to dramatically alter the skewed gut microbiota and reverse colitis in model mice associated with an increase of intestinal γδT cells. Through 16S rDNA sequencing, we showed that LFS-01 can selectively suppress enteric pathogens such as *Escherichia–Shigella* and *Helicobacter* whereas the protective strains including *Lactobacillus* and *Lachnospiraceae* were significantly expanded after LFS-01 treatment. Interestingly, we demonstrated that LFS-01 administration can significantly promote the IL-17+γδT cells in model mice in response to the expanded *Lactobacillus*. We verified that the intracellular components of *Lactobacillus* can stimulate the growth of IL-17+γδT cells upon preincubation. The increased IL-17A after LFS-01 treatment in turn recovers the disrupted occludin subcellular location and protects the epithelial barrier in the colon of model mice. Remarkably, LFS-01 does not show apparent toxicity to animals and we demonstrated that LFS-01 also exerts strong protective effects in TNBS-induced colitis rats. Therefore, LFS-01 holds great promise for the treatment of inflammatory bowel disease (IBD) and warrants translation for use in clinical trials. Our work provided a new avenue for the treatment of IBD based on the strategy of harnessing intestinal symbiosis.

## Introduction

The human gastrointestinal (GI) tract harbors the highest density and complexity of microbial organisms in the body, known as the microbiota (Backhed et al., 2005; Qin et al., 2010; Blumberg and Powrie, 2012; Okai et al., 2016). Inflammatory bowel disease (IBD), an immunological disorder which consists of two clinical phenotypes: ulcerative colitis (UC) and Crohn’s disease (CD), has arisen as a major threat to human health globally in recent decades (Baumgart and Carding, 2007). Both UC and CD can cause prolonged inflammation of the digestive tract and result in disease-related mortality. Over 2 million residents in the United State, 2.5 million residents in Europe and over 2 million residents in China are estimated to have IBD (Ananthakrishnan, 2015; Kaplan, 2015). Moreover, the incident rates of IBD keep rising around the world. It has been well recognized that the dysbiosis of gut microbiota may disrupt intestinal homeostasis and eventually lead to the pathogenesis and development of IBD (Dalal and Chang, 2014; Kostic et al., 2014). Unfortunately, there is currently no known cure for IBD and effective treatments are still lacking.

There have been accumulating evidences suggesting that commensal microbiota that populates epithelial surface can function as a potent immunomodulator for intestinal mucosal T cells such as γδT cells (Belkaid and Hand, 2014; Benakis et al., 2016). Importantly, γδT cells is a major producer ofinterleukin-17A (IL-17A, also named as IL-17), a hallmark cytokine that contributes to intestinal immunopathology (Cua and Tato, 2010). The roles for IL-17A in the gut mucosa during inflammation remain controversial. Indeed, recent studies suggested that IL-17A plays a protective role for maintaining barrier function during intestinal damage (Kinugasa et al., 2000; O’Connor et al., 2009). For instance, it has been implicated the neutralization of IL-17A significantly increases the intestinal tissue damage in dextran sodium sulfate (DSS) induced murine model of colitis (Ogawa et al., 2004). In particular, a recent study has implicated that IL-17A can promote epithelial barrier function by regulating the tight junction protein occluding through the IL-17R adaptor protein Act-1 on epithelial cells in DSS-induced colitis model (Lee et al., 2015). Hence, IL-17A-producing γδT cells can protect the epithelial barrier against excessive gut permeability during intestinal injury.

Sulforaphene (LFS-01) is the major chemical constituent of *Raphanus sativus* (also named as *Lai Fu Zi* in traditional medicine book), a medicinal herb used in traditional Chinese medicine since the Song dynasty to treat coughing and food stagnation for over a 1000 years. In this study, for the first time, we showed that oral treatment of LFS-01 can reverse the development of DSS-colitis by selectively suppressing the harmful enteric pathogens and promoting the *Lactobacillus* population accompanied with a significant increase of IL-17^+^γδT cells. Further, we revealed that *Lactobacillus* population can directly stimulate the growth of γδT cells upon preincubation mediated through TLR2. Noteworthy, LFS-01 does not show apparent toxicity to mice and therefore may present as a rather promising therapy to treat IBD in clinical practice.

## Materials and Methods

### Animal Experiments

Male WT C57BL/6J mice were obtained from the Experimental Animal House of Dalian Medical University, China, where they were maintained under stress free and specific pathogen-free (SPF) conditions, at 22 ± 3°C and 70% ± 5% relative humidity, under 12-h cycles of light and darkness. Food and water were provided *ad libitum* before experiments. The animals were allocated randomly into three groups with seven animals in each group. We want to remind the reader that the gut microbiota can be largely influenced by cage effects. In our experiments, we used a large cage (size: 55 cm ^∗^ 40 cm ^∗^ 20 cm) to house each group of animals (seven mice per group). We recognize that the gut microbiota of the animals housed in one cage are likely to be concordant due to cage effects. The animal experimental procedures were approved by the Medical Ethics Committee of local Government and University (SYXK2015-0002).

### Chemical Compounds

The LFS-01 used in the present study was purchased from Hangzhou Tianhong Biotechnology with >97% purity (HPLC grade) identified by (Backhed et al., 2005) H-NMR and Mass-spectra. We also purchased standard sample from Santa Cruz Biotechnology for comparison purpose.

### DSS-Induced Colitis, LFS-01, 5-ASA, and FITC-Dextran Oral Administration

To induce acute colitis, 6-8 weeks old mice were given drinking water containing 3.0% (w/v) DSS (MP Biomedicals, MW: 360,000–500,000) *ad libitum* for 7 days and distilled water for one additional day before sacrifice. Mice were monitored for weight and signs of diseases. Disease activity index (DAI) was scored based on a non-blinded fashion. To detect gut permeability, the animals were gavaged with FITC-Dextran (4 kDa, Sigma–Aldrich) as described before (Tang et al., 2015) 3 h prior to fluorometric analysis of FITC fluorescence in plasma. LFS-01 or 5-ASA (Energy Chemical) were first dissolved in ethanol (70%) and then diluted in saline water. The solutions were administrated to DSS-drinking mice with a gastric tube by the dose of 80 mg/kg-body weight. Sulforaphene (LFS-01) was administered to the animals starting from day-1 (the very day that DSS was administered to induce colitis) to day-7. The animals were sacrificed at day-8 for further analysis.

### Histopathological Analysis, Myeloperoxidease (MPO), and Cytokines Measurement

The DAI of mice was evaluated as previously described (Gonzalez-Rey et al., 2006). After mice were sacrificed, the colon was extracted, cleaned, and weighed. The colonic tissue samples were preserved in buffered formalin, embedded in paraffin, cut into 5 μm sections and stained with hematoxylin and eosin (H&E) for histopathological analysis. The rest of the colonic tissue was homogenized in a Greenburger buffer supplemented with protease inhibitors (complete Mini EDTA free, Roche). After sonication for 10 s, the suspension was centrifuged at 8000 × *g* for 20 min at 4°C. The supernatant was used to quantify the myeloperoxidease (MPO) activity and the levels of cytokines using commercial ELISA kits (USCN, United States) following the manufacturer’s protocol. Peripheral blood was taken and centrifuged immediately at 1500 × *g* for 15 min at 4°C to obtain serum. The concentrations of serum cytokines were also assayed by ELISA (USCN, United States) according to the manufacturer’s instructions.

### DNA Isolation from Colonic Contents

The metagenomic DNA in the colon content of mice in each group was extracted by the QIAamp DNA stool mini kit (Qiagen, Germany). The purity and concentration of the metagenomic DNA were measured by NanoDrop 2000 spetrophotometer (Thermo, United States).

### Library Construction

The V3–V4 region of 16S rDNA (representing bacteria) and the internal transcribed spacer regions 2 (ITS2, representing fungi) were amplified with the primers. Primer sets were modified with Illumina adapter regions for sequencing on the Illumina GAIIx platform, and reverse primers were modified with an 8-bp Hamming error-correcting barcode to distinguish among samples. The DNA template (100 ng) was combined with 5 μL PCR buffer, 1 μL dNTPs, 0.25 μL HotStarTaq^®^ Plus DNA Polymerase (Qiagen), and 2.5 pmol of each primer in 50 μL total volume. Reactions consisted of an initial step at 95°C for 5 min; 25 (16S rDNA) or 38 cycles (ITS2 rDNA) of 94°C for 45 s, 55°C for 45 s, and 72°C for 60 s; and a final extension at 72°C for 10 min. DNA products were checked by 1.5% (w/v) agarose gel electrophoresis in 0.5 mg/mL ethidium bromide and purified with the Qiaquick gel extraction kit (Qiagen).

### Bioinformatics Analysis

Sequences of the V3–V4 region of 16S rDNA and ITS2 in mouse intestine were detected using an Illumina HiSeq 2000 platform (reconstructed cDNA sequence: 2 × 150 bp, Novogene Bioinformatics Technology Co. Ltd, Beijing). Ribosomal Database Project (RDP) Classifier 2.8 was used for taxonomical assignment of all sequences at 50% confidence after the raw sequences were identified by their unique barcodes. OTUs present in 50% or more of the fecal samples were identified as core OTUs. PLS-DA of core OTUs was performed using Simca-P version 12 (Umetrics), and a heat map was generated with Multi-Experiment Viewer (MeV) software to visualize and cluster the fungal community into different groups. Community diversity was measured by the Shannon–Weiner biodiversity index (Shannon index). This work was conducted by Novogene Institute (Beijing, China).

### Cell Isolation from Colonic Lamina Propria (cLP)

Colons were removed from the control, DSS-administered or LFS-01 treated mice followed by incubating in a 37°C water bath in cell dissociation solution made with HBSS, 5 mM and HEPES (Solarbio) so that the epithelial cells of mouse colon were stripped. Supernatant was discarded and colonic tissue was then incubated in a digestion cocktail containing HBSS, 10% FCS (Gibco), 1 mg/ml collagenase type IV, 0.5 mg/ml DNaseI, and 0.5 mg/ml dispase (all from Sigma–Aldrich) in a 37°C water bath. The digested tissue was processed through a 70 mm (Falcon) filter and washed before lymphocytes were separated using a percoll gradient (Solarbio, 40–80%) and re-suspended in complete RPMI (Hyclone) supplemented with 10% FCS (Gibco), 1% HEPES, 50 Mm 2-mercaptoethanol (Gibco), 1% sodium pyruvate, and penicillin and streptomycin (Gibco). Cells were centrifuged at 12000 r.p.m. for 10 min at 4°C, washed three times with D-Hank’s solution and stored on ice.

### Flow Cytometry Analysis

For IL-17A staining, cells were stimulated with 2 μl/ml Cell Stimulation Cocktail (500×, plus protein transport inhibitors, eBioscience) in the presence of 100 μl Intracellular Fixation &Permeabilization Buffer Set (eBioscience) Golgi-plug (eBioscience) for 12 h in complete medium. Surface staining was then performed in the presence of Fc-blocking antibodies (Albumin, bovine, fraction V-Coolaber) and using αCD4 (Anti-Mouse CD4 FITC, 11-0041, eBioscience), α-γδTCR (Anti-Mouse gamma delta TCR PE, 12-5711, eBioscience), α-TLR2 (Anti-Mouse CD282 eFluor^®^660, 50-9021, eBioscience). Cells were then fixed and permeabilized with cytofix-cytoperm kit (BD) followed by intracellular staining using antibodies against IL-17A (Anti-Mouse /Rat IL-17A APC, 17-7177, eBioscience). All samples were collected (Accuri C6, BD Bioscience, United States) and data were analyzed with Flow Plus 1.0.264.15 Software.

### Immunofluorescence Microscopy

Distal colons were flushed with PBS, embedded in Tissue-Tek O.C.T. compound (SAKURA Finetechnical Company) in cryomolds, and snap frozen in liquid nitrogen for cryosectioning. Cryosections were prepared on a Leica Cryostat (Leica Microsystems) at -20°C in 8 μm thickness. Sections were mounted on glass slides and fixed in 100% ethanol at 4°C for 30 min followed by 3 min of 20°C acetone fixation at room temperature. The slides were washed in PBS and blocked in FBS and goat serum for 1 h in room temperature. The tissue sections were stained with a monoclonal occludin antibody sc-5562 (Santa Cruz Biotechnology) at 4°C overnight. After washing in PBS, the sections were stained with a goat anti-rabbit IgG Alexa Fluor 488 conjugated secondary antibody (Fcmacs) for 60 min at room temperature. After washing in PBS, the tissue sections were treated with DAPI (Millipore) for 5 min and covered with a coverslip. Fluorescence was observed with a microscope (DM4000B; Leica, Germany) equipped with a digital camera at 40× magnification.

### RNA Isolation and Real-Time Quantitative PCR (qPCR)

To analyze the mRNA expression levels of genes, total RNA in mouse colon tissues were extracted with the RNeasy mini Kit (Qiagen, Hilden, Germany). The complementary DNA (cDNA) was synthesized using the AffinityScript Multiple Temperature cDNA synthesis Kit (Stratagene, La Jolla, CA, United States) according to the supplier’s protocol. cDNAs obtained by reverse transcription were used to determine mRNA expression levels of Occludin, IL-17A, Act-1, TLR2, and Dectin-1 by the specific primers. The reactions were run on an ABI StepOne Plus Sequence Detection System (Applied Biosystems). The reaction mixture (10 mL) comprised 4.5 mL FastStart Universal SYBR Green Master (Roche, Mannheim, Germany), 0.5 mL of each primer 30 mM, 2.5 mL of sterile distilled water, and 2 mL of DNA template (100 ng/mL). Each sample was run in triplicate, and the mean Ct was determined from the three runs. Relative mRNA expression was expressed as ΔCt = Ct (target genes) - Ct (calibrator). GAPDH housekeeping gene expression was used as calibrator after verification of its stability under the experimental conditions. Further, relative mRNA expression was calculated as ΔΔCt = ΔCt (test group) - ΔCt (control group). Lastly, the relative gene expression levels were converted and expressed as fold difference (=2^-Δ ΔCt^).

### The Isolation of Colon Contents

The colons were removed from mice after sacrifice. Next, 5 ml of PBS (pH∼7.2) containing 0.02% sodium azide was passed through the colon in order to collect the colon contents. The washout contents were centrifuged at 6000 r.p.m. for 30 min at 4°C and the supernatant was harvested. Proteinase inhibitor PMSF was added at the concentration of 1 mmol/L. Lastly, the samples were stored at -80°C for further analysis.

### γδT Cell Isolation from Colon and Spleen of Animals

The colons of mice were incubated in a 37°C water bath in cell dissociation solution made with HBSS and HEPES (Solarbio) to strip the epithelial cells. Supernatant was discarded and colonic tissue was then incubated in a digestion cocktail containing HBSS, FCS (10%, Gibco), type IV1 collagenase (1 mg/ml), DNaseI (0.5 mg/ml), and dispase (0.5 mg/ml, all from Sigma–Aldrich) in a 37°C water bath. After that, the digested tissue was processed through a 70 mm filter (Falcon) and washed before lymphocytes were separated by the methods used previously. γδT cells were then purified from the spleens of mice using the Mouse TCRγδ + T-cell Isolation Kit and the miniMACSTM (MiltenyiBiotec, Germany).

### γδT Cells Cultured with *Lactobacillus* Breves

Cells were put into single-cell suspensions at 1–2 × 10^6^ cells/ml and were plated for a final volume of 200 μl and re-stimulated with media alone or with media and bacterial components. A total of 1 ml of the bacterial consortium, or culture broth of *Lactobacillus* breves DM9218, was sonicated to release the cellular components. Subsequently, the mixture was centrifuged at 8,000 rpm for 2 min, and different volumes of the supernatant were added to the single-cell suspension after filtration by a 0.2 μm-filter. After co-culture for 20 h, the IL-17A level in the cell culture supernatant was detected by ELISA (USCN, United States).

### Western Blot

The total protein was extracted from colon tissues of animals. Equal amounts of proteins were fractioned with sodium dodecylsulfate polyacrylamide gel electrophoresis (SDS-PAGE) followed by electrophoretic transfer of proteins onto nitrocellulose membranes. The blots were probed with antibodies against Occludin, Act-1, IL-17RC GADPH (USCN, United States), and followed by incubation with secondary antibodies conjugated with horseradish peroxidase (HRP, USCN, United States). The immune complexes were detected with a WesternBright^TM^ ECL Western Blotting HRP Substrate kit and analyzed with image lab software (Bio-Rad, United States).

### Statistics

All data were evaluated as mean ± SEM. Statistical analysis of the quantitative multiple group comparisons was performed using the one-way analysis of variance (ANOVA) followed by Tukey’s test; whereas pairwise comparisons were performed using the *t*-test by GraphPad Prism 5 (Graph Pad Software, La Jolla, CA, United States). Results were considered to be statistically significant with *p* < 0.05.

## Results

### Oral Administration of LFS-01 Reverses DSS-Induced Acute Colitis

**FIGURE 1:**
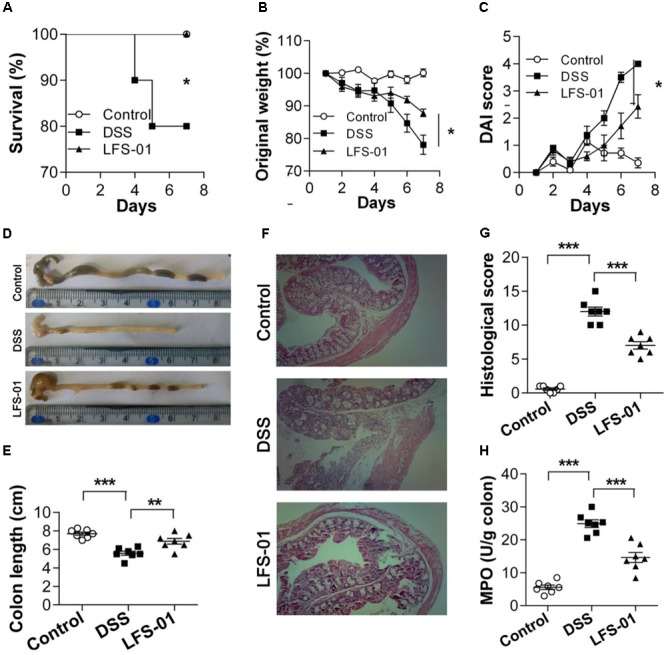
Oral administration of sulforaphene (LFS-01) reverses dextran sodium sulfate (DSS)-induced colitis in model mice. Oral treatment of LFS-01 was assessed by **(A)** survival rate; **(B)** change of original weight; **(C)** DAI score. **(D,E)** The colon length was determined after sacrifice. **(F)** H&E-stained results for the sections of mouse colon. **(G)** Histopathological analysis for the H&E-stained sections. **(H)** The MPO activity of colonic tissue was detected by ELISA following the manufacturer’s protocol. All data were evaluated as mean ± SEM (*n* = 7). Statistical analysis of the quantitative multiple group comparisons was performed using the one-way analysis of variance (ANOVA) followed by Tukey’s test, ^∗^*p* < 0.05, ^∗∗^*p* < 0.01, ^∗∗∗^*p* < 0.001.

First, we assessed whether LFS-01 oral treatment could reverse acute colitis induced by DSS. In line with prior reports (Wirtz et al., 2007), mice subjected to 7 days’ administration of DSS supplemented in drinking water developed an acute colitis characterized by bloody diarrhea, ulcerations and infiltrations with granulocytes and showed severe weight loss resulting in a mortality of 20% (**Figures 1A,B,D,F**). In contrast, mice treated with 80 mg/kg dose of LFS-01 after DSS administration recovered the lost body weight with a healthy appearance and a survival rate of 100%, similar to the control group treated with vehicle (**Figures 1A,B**). All mice were sacrificed on day 7 for macroscopic and histopathological analysis of the colon by a board-certified pathologist. Macroscopic examination of animal colons obtained 7 days after DSS induction demonstrated severe hyperemia and inflammation compared with control group whereas animals treated with LFS-01 exhibited significantly reduced swelling, inflammation and ulceration, quantified by DAI score (**Figures 1C,D**). Moreover, we observed a statistically significantly reduction of colon length and a significant increase of colonic myeloperoxidase (MPO) activity in the DSS-induced group compared to the control group. In contrast, the animal group treated with LFS-01 exhibited a striking improvement of both colon length and MPO (**Figures 1D,E,H**). We evaluated the microscopic damage by histopathology analysis of the colon tissue sections at the end of dosing period. We observed an extensive destruction of the colon mucosal layer in the DSS-induced group characterized by increase in the thickness of the muscular layer, ulceration, epithelial cell loss, reduction of the density of the tubular glands and disseminated fibrosis. Remarkably, LFS-01 treatment significantly recovered the microscopic damage as compared to the DSS-treated group (**Figures 1F,G**). In addition, animals treated with LFS-01 exhibited no incidence of diarrhea over the course of treatment (data not shown). These data provide strong evidences that LFS-01 oral treatment can reverse the course of DSS-induced acute colitis.

### LFS-01 Treatment Profoundly Alters the Gut Microbiota in DSS-Induced Model Mice

**FIGURE 2:**
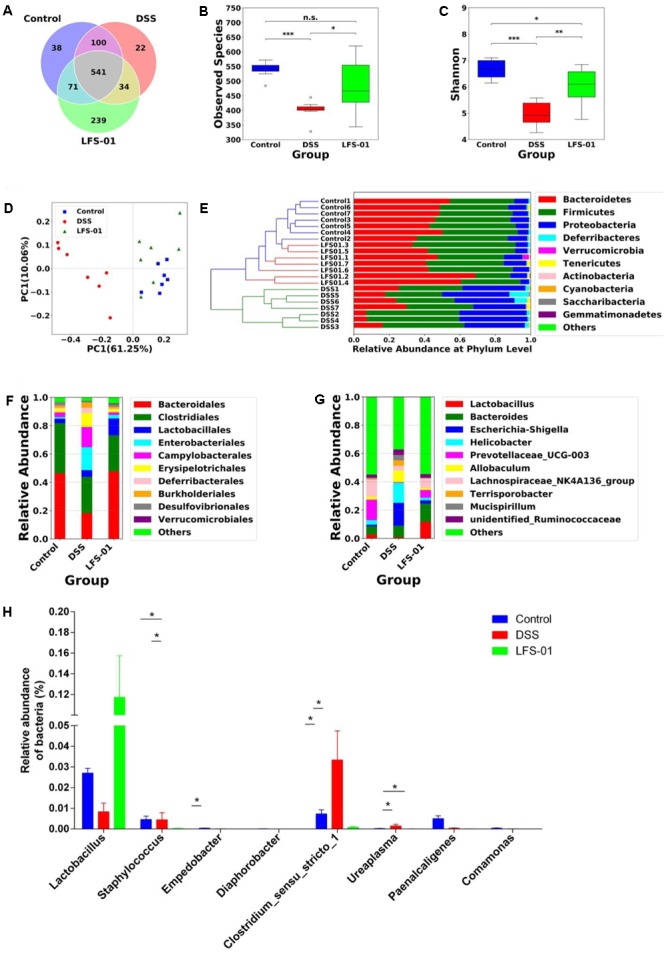
Sulforaphene treatment modulates intestinal bacterial microbial composition in model mice. **(A)** Venn diagram of shared and independent bacterial OTUs in different experimental groups (*n* = 7). **(B,C)** Comparison of the observed bacterial species and Shannon index of different groups. **(D)** Principal Coordinate Analysis (PCoA) based on weighted Unifrac distances among different samples. PC1 and PC2 account for 71.31% of the variation. **(E)** Unweighted Pair-group Method with Arithmetic Mean (UPGMA) clustering of bacterial microbial composition at phylum level in different samples. **(F,G)** The composition of bacterial microbial composition in different experimental groups at order and genus levels, respectively. **(H)** The relative abundance of bacterial groups at genus level between groups tested by means of one-way ANOVA followed by Tukey’s test. ^∗^*p*-value < 0.05.

The commensal microbiota plays an important role in the pathogenesis of intestinal inflammation. The dysbiosis of both bacterial and fungal species has been implicated in IBD patients as well as in animal models of experimental colitis (Sokol et al., 2016). We therefore wanted to examine if LFS-01 treatment can alter the gut microbial composition using culture-independent analysis based on the 16S rDNA and ITS2 genes sequencing experiments. First, the 16S rDNA sequencing results revealed that the overall OTUs of intestinal bacteria and the alpha diversity exhibit significant differences between animal groups (**Figures 2A–C**). Compared with the control group, the observed bacterial species as well as the alpha diversity were reduced in DSS-treated mice. LFS-01 treatment altered the bacterial structure of mouse intestine as evaluated by principal coordinate analysis (PCoA, **Figure 2D**). We observed a significantly decreased abundance of *Bacteroidetes* (phylum), *Spirochates* (phylum), *Bacteroidales* (order), *Clostridales* (order) and *Lactobacillus* (genera) in DSS-treated group compared to the control group (**Figures 2E–G**). Moreover, we found an elevated abundance of *Proteobacteria* (phylum), *Enterobacteriales* (order), *Escherichia–Shigella* (genera), and *Helicobacter* (genera) in DSS-treated mice (**Figures 2E–G** and Supplementary Figures S1–S3). Strikingly, LFS-01 administration recovered these skewed bacterial groups caused by DSS treatment, particular for the strains of *Escherichia–Shigella* and *Helicobacter* (Supplementary Figures S1, S2). More interestingly, LFS-01 treatment significantly promoted the colonization of *Lactobacillus* and *Lachnospiraceae* (**Figures 2G,H** and Supplementary Figures S2, S3). Of note, prior literatures have well established that both *Lactobacillus* and *Lachnospiraceae* are protective strains for IBD.

**FIGURE 3:**
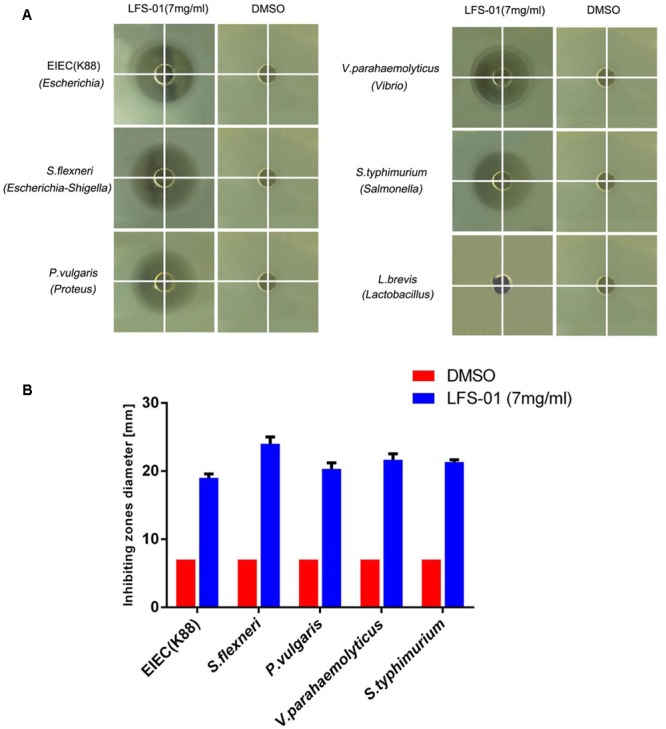
Sulforaphene is protective against various enteric pathogens yet with no inhibitory effects on *Lactobacillus*. **(A)** Radial diffusion assay of enteric pathogens, *Lactobacillus*, and conditioned medium. **(B)** Column diagram of inhibiting zones diameter for radial diffusion assays.

To evaluate the suppression of LFS-01 against enteric pathogens, we selected numerous bacterial species for radial-diffusion assay test including *E. coli*/*K88* (*Escherichia*), *S. flexneril* (*Escherichia*–*Shigella*), *P. vulgaris* (Proteus), *V. parahaemolyticus* (Vibrio), and *S. typhimurium* (Salmonella). These strains were selected because they may be associated with the pathogenesis of IBD. For instance, prior research has established that *Escherichia coli* (Rhodes, 2007), *Escherichia*–*Shigella* (Morgan et al., 2012), *Vibrio* and *Salmonella* (Gradel et al., 2009) are either significantly increased in the GI of IBD patients or closely associated with the pathogenesis of IBD. In brief, the application of LFS-01 on agar generated a clear zone of inhibition of all five enteric pathogens growth (7 mg/ml). Interestingly, we found that LFS-01 barely has any observable effects on the growth of *Lactobacillus* strain (*L. brevis*) in the same concentration (**Figure 3** and Supplementary Table S1). Noteworthy, *L. brevis* has been documented as a probiotic strain commonly found in the intestine of animals or human.

Furthermore, we detected 37 core fungal OTUs overlapped between three animal groups and we found 165 excessive OTUs in DSS-treated group (Supplementary Figure S4a). We demonstrated that LFS-01 treatment reconstitutes the skewed fungal structure evidenced by PCoA and taxa heatmaps (Supplementary Figure S4b). We found that LFS-01 treatment significantly reduced the abundance of more than 17 genera of fungal microbiota such as *Meyerozyma, Kazachistania, Mortierella, Rhodosporidum, Hansfordia, Curvularia, Fusarium, Khuskia*, and *Malassezia* etc. which were elevated in DSS-treated group (Supplementary Figures S4c–e).

### LFS-01 Treatment Enhances the Production of IL-17+γδT Cells after Intestinal Injury

**FIGURE 4:**
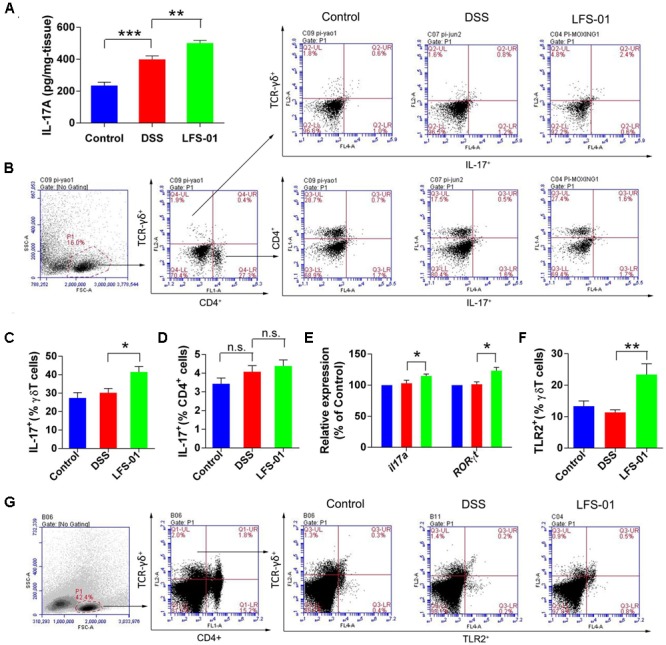
Sulforaphene administration promotesIL-17^+^ γδT cells in cLP of colitis mice. **(A)** The IL-17A protein concentration in each animal group was detected in supernatants of colonic tissue homogenates by ELISA. **(B)** Representative flow cytometry plots of CD4^+^ T cells identified using side scatter (SSC) and forward scatter (FSC) plots (left panel) and TCR-γδ^+^expression. Representative flow cytometry plots of Th17 cells (CD4^+^IL-17^+^) and γδT17 cells (TCR-γδ^+^IL-17^+^)in the LP of mouse colon (right panel). **(C)**. Percentage of γδT17 cells in γδT cells of cLP in mice (*n* = 7 mice per group). **(D)** Percentage of Th17 cells in CD4^+^ T cells of cLP in mice (*n* = 7). **(E)** The relative expression levels of *il17a* and *RORγt* in colon tissues of mice detected by quantitative real-time PCR (qPCR). **(F)** Quantification of TLR2 expressing γδT cells (TLR2^+^TCR-γδ^+^) in the cLP of mice in different groups. **(G)** Representative flow cytometry analysis of TLR2 expressing γδT cells. Error bars represent mean ± SEM (*n* = 7). Statistical analysis of the quantitative multiple group comparisons was performed using the one-way analysis of variance (ANOVA) followed by Tukey’s test, ^∗^*p*<0.05, ^∗∗^*p*<0.01, ^∗∗∗^*p*<0.001; n.s., not significant.

We observed that the mucosal level of IL-17A was increased in both DSS and LFS-01 treated animal groups compared with the control group (**Figure 4A**). Yet, the elevation levels of IL-17A in LFS-01 treated group were significantly higher than those of DSS treated group. The adaptive T helper 17 (Th17) cells (Conti et al., 2009) and γδT cells (Roark et al., 2008) have been regarded as two major producers of IL-17 against pathogens at mucosal surface. Hence, we employed flow cytometry analysis to detect the specific contribution of Th17 cells and γδT cells to IL-17A production in our experiments. We found that IL-17 producing γδT cells were markedly increased in LFS-01 treatment group compared to DSS-treated group and the control group (**Figures 4B–D**). Moreover, we demonstrated that the mRNA expression levels of IL-17A-encoding gene and the IL-17A-specific transcription factor RORγT were also elevated as compared to the DSS-treated group and the control group (**Figure 4E**). Furthermore, we evaluated the production of inflammatory mediators upon DSS treatment and LFS-01 administration. We found an elevated protein expression of inflammatory cytokines including IL-6, IL-23, IL-1β, and TNF-α in the mucosa of colitis mice compared to the control group (Supplementary Figure S5). As expected, LFS-01 treatment dramatically reduced the production of these inflammatory cytokines. Furthermore, in contrast, we found that the anti-inflammatory cytokine IL-10 and IL-22 were increased in LFS-01 administrated group compared to the DSS-treated group (Supplementary Figure S5). Prior studies have shown that IL-17+γT cells can selectively expand in response to microbial secreted products through expressing of TLR2 and dectin-1 (Mokuno et al., 2000; Martin et al., 2009). Therefore, we examined the specific contribution of TLR2 and Dectin-1 expressing γT cells in three animal groups. Importantly, flow cytometry analysis showed that the percentage of TLR2+γT cells, instead of Dectin-1+γT cells, were significantly enhanced upon treatment of LFS-01 (**Figure 4G** and Supplementary Figure S6). Hence, our results implicate that LFS-01 treatment enhances the production of IL-17+γδT cells after intestinal injury in response to the secreted products of microbiota mediated through TLR2.

### LFS-01 Treatment Restores Intestinal Epithelial Permeability by Modulating the Subcellular Localization of Occludin Acting through the Axis of IL-17 and Act-1

**FIGURE 5:**
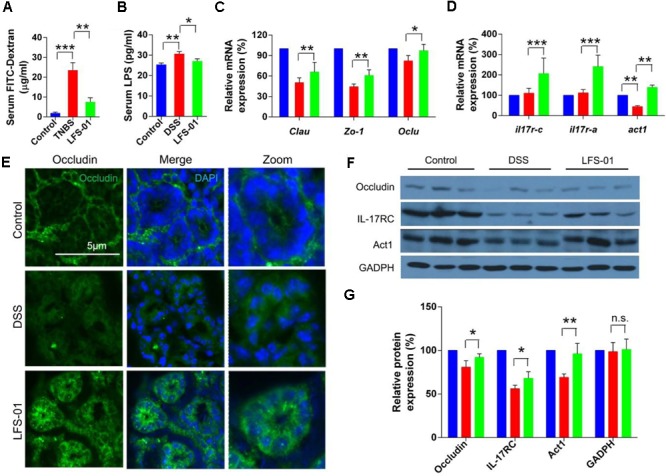
Sulforaphene administration decreases gut permeability of colitis mice by regulating IL-17-Act1-occludin axis. **(A,B)** Detection of FITC-dextran and LPS in serum of mice by ELISA. **(C)** The relative expression levels of genes encoding tight junction proteins in colon tissues of mice detected by Qpcr. **(D)** The relative expression levels of genes encoding IL-17RC, IL-17RA, and Act1 in colon tissues of mice detected by Qpcr. **(E)** Representative immunofluorescence images of occluding (green) and DNA (clue) of distal colon segments from mice of different groups. The third column represents a magnified image. **(F)** The expression of IL-17RC, Act1, and occludin detected by Western blot (WB) with GADPH as internal control. **(G)** The relative expression levels of IL-17RC, Act1, and occluding deduced from the WB results. error bars represent mean ± SEM (*n* = 7). Statistical analysis of the quantitative multiple group comparisons was performed using the one-way analysis of variance (ANOVA) followed by Tukey’s test, ^∗^*p* < 0.05, ^∗∗^*p* < 0.01, ^∗∗∗^*p* < 0.001; n.s., not significant.

The excessive mucosal permeability is often observed after intestinal injury and thus used as a critical pathological feature for IBD patients. We wanted to determine if LFS-01 treatment could reverse the excessive gut permeability in DSS-induced colitis model. The gut epithelial permeability was quantified by orally administering FITC-dextran to animals in each group on day 7 and measuring the amount present in the colon serum. The diffusion of FITC-dextran across the gut epithelium in the DSS-induced group was significantly higher compared to those in the control group, suggesting an abnormal barrier function during DSS-induced intestinal injury (**Figure 5A**). Remarkably, LFS-01 treatment corrected the excessive gut permeability and significantly reduced the diffusion of FITC-dextran across the gut epithelium compared to the model group (**Figure 5A**). The amount of serum LPS level was also restored in the LFS-01 treatment group (**Figure 5B**). Furthermore, in each group of animals, we assessed the transcript expression level of claudins, zonula occludens-1 (ZO-1), and oculudins, three proteins which forms the tight junction complex for regulating intestinal permeability and the epithelial paracellular pathway. As expected, transcripts of *Clau* and *Zo-1* are significantly downregulated in DSS-induced colitis group compared to the control group whereas in LFS-01 treatment group, the transcripts level of both proteins were recovered. We didn’t observe significant change of *Ocln* transcript level between DSS-induced colitis group and the control group (**Figure 5C**). However, we observed that the subcellular localization of oculudins was altered in the epithelial cells of the colonic crypts in the DSS-induced colitis group compared to the control group. Namely, in the control group, oculudins were mainly localized on the apical surface of the epithelial cells as demonstrated in the cross sectional confocal images (**Figure 5E**). In contrast, the gut epithelial cells in the DSS-induced colitis group exhibited diffused occludin staining that appeared to extend into cytoplasm as compared to the control group (**Figure 5E**). This is consistent with prior studies that the internalization of occludin from the tight junction contributes to increased intestinal permeability during intestinal injury. Interestingly, the disruption of subcellular localization of occludin was dramatically corrected in the LFS-01 orally administered group (**Figure 5E**). Importantly, a very recent study (Lee et al., 2015) has suggested that IL-17 (also named as IL-17A), a hallmark cytokine with controversial role in the gut mucosa, regulates occludin subcellular location and actually protects epithelial barriers by signaling through the IL-17 receptor adaptor protein Act-1. We therefore wanted to evaluate if LFS-01 treatment can impact IL-17A and Act-1. Very interestingly, we found that both the transcript and protein expression level of IL-17A and Act-1 were enhanced after LFS-01 treatment compared to DSS-induced colitis group (**Figures 5D,F,G**). These results strongly indicate that LFS-01 is able to restore abnormal mucosal permeability after intestinal injury by modulating disrupted occludin subcellular location acting through the signaling axis of IL-17A and Act-1.

### *Lactobacillus* Directly Stimulates the Growth of γδT Cells

**FIGURE 6:**
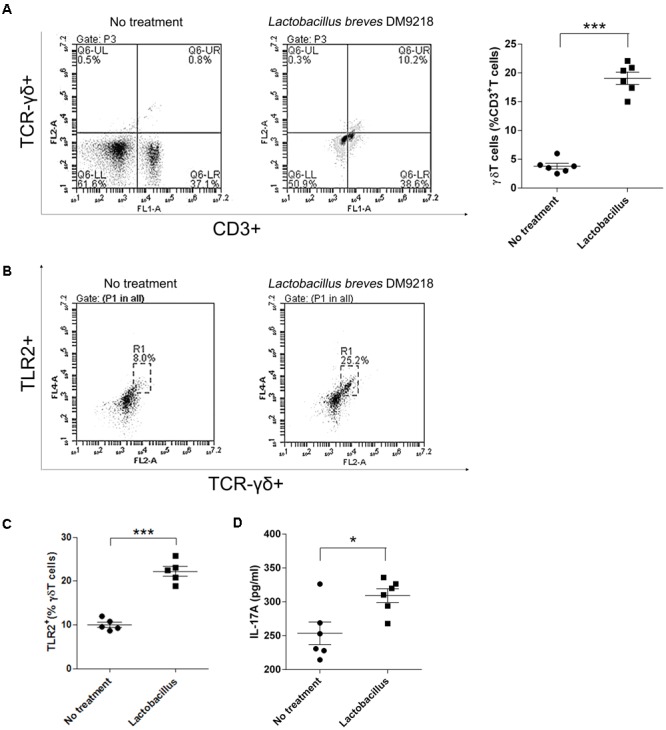
*Lactobacillus* stimulates γδT cells in colonic lamina propria (cLP) of colitis mice. **(A)** Representative flow cytometry plots and evaluation of the percentage of γδT cells among cLP cells treated or un-treated with 30 μl of the *Lactobacillus* breves DM9218 *in vitro*. All values are mean ± SEM (*n* = 6). The non-parametric *t*-test was performed by the assistant of GraphPad Prism 5; ^∗^*p* < 0.05. **(B)** Effects of the *Lactobacillus* spp. on TLR2 expression and IL-17A secretion of purified splenic γδT cells. Representative flow cytometry plots and of TLR2+ γδT cells among γδT cells treated or un-treated with 30 μl of the *Lactobacillus* culture supernatent *in vitro*. The γδT cells were purified by MACS from the spleen of C57BL/6J mice at day 7, which belonged to the control group. **(C)** Evaluation of the percentage of TLR2+γδT cells among γδT cells treated or un-treated with 30 μl of the *Lactobacillus* culture supernatent *in vitro*; IL-17A concentration in the supernatant of purified γδT cells treated or un-treated with 30 μl of the *Lactobacillus* culture supernatent *in vitro* detected by ELISA. All values are mean ± SEM (*n* = 6). The non-parametric *t*-test was performed by the assistant of GraphPad Prism 5; ^∗^*p* < 0.05.

As abovementioned, we observed that LFS-01 treatment can promote γδT cells in model mice accompanied by the expanded *Lactobacillus*. Therefore, we wanted to examine if *Lactobacillus* strains can directly stimulate the growth of γδT cells. Interestingly, we found that preincubation of colonic lamina propria (cLP) cells with *Lactobacillus* breves DM9218 can significantly increase the percentage of isolated γδT cells (**Figure 6A**). In our experiment, we used *Lactobacillus* breves DM9218, a strain originally isolated by our lab from Chinese fermented food. Further studies are warranted to use more strains of *Lactobacillus* to confirm the findings. Moreover, we revealed that *Lactobacillus* spp. can induce TLR2 expression and IL-17A secretion of purified splenic γδT cells (**Figures 6B,C**). These evidences strongly support our hypothesis that *Lactobacillus* strains may stimulate the growth of γδT cells in the colon.

### Water-Soluble Formulation of LFS-01 Exerts Excellent Therapeutic Potentials

**FIGURE 7:**
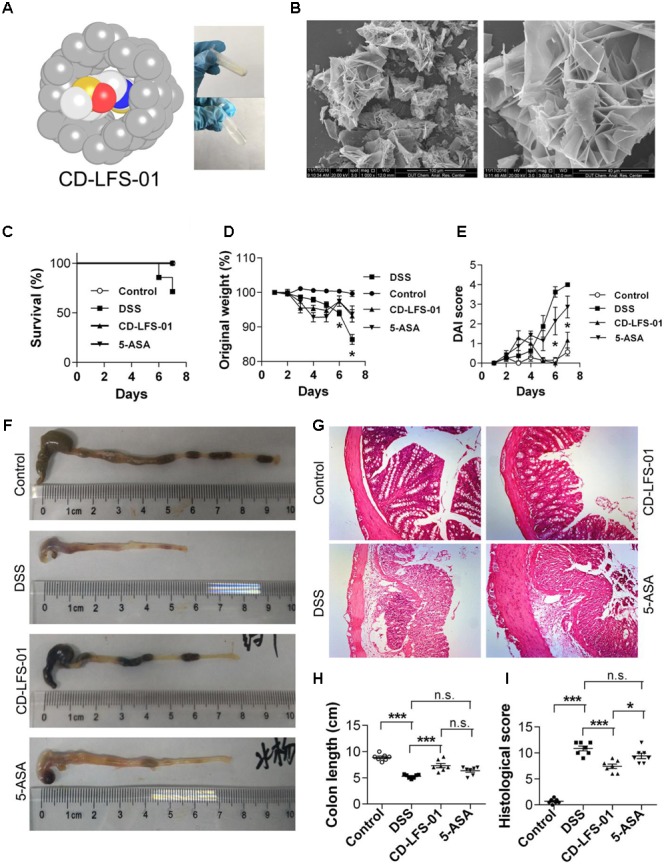
Water-soluble formulation CD-LFS-01 (also named as Cyclone-01) exhibited superior protective effects on colitis mice than 5-ASA. **(A)** Molecular model of LFS-01 encapsulated in α-Cyclodextrin (α-CD) and CD-LFS-01 contained in a vial (in powder and dissolved in 0.5 ml water, respectively). **(B)** The scanning electron microscopy (SEM) data for water-soluble formulation CD-LFS-01. The clinical outcome of CD-LFS-01 in DSS-induced colitis model was assessed by **(C)** survival rate; **(D)** change of original weight and **(E)** DAI score; **(F)** the colon length was determined after sacrifice. **(G)** The sections of mouse colon was H&E-stained (Magnification, 200×). **(H)** Histopathologic analysis was performed in the H&E-stained sections. **(I)** Colon length in different groups. All data were evaluated as mean ± SEM (*n* = 7). Statistical analysis of the quantitative multiple group comparisons was performed using the one-way analysis of variance (ANOVA) followed by Tukey’s test, ^∗^*p* < 0.05, ^∗∗∗^*p* < 0.001, n.s., not significant.

To enhance the oral bioavailability and therapeutic properties of LFS-01, we prepared a nanoparticle encapsulated form of LFS-01 by α-Cyclodextrin (named as CD-LFS-01 or Cyclone-01^®^, **Figure 7** and Supplementary Figures S7, S8). This nanoparticle encapsulated form can also increase the stability and colon-targeting properties of LFS-01 or its structural analogs. We tested the therapeutic efficacy of CD-LFS-01 in DSS-induced colitis rodent model with 5-aminosalicylic acid (5-ASA) as a control (**Figure 7**). Of note, 5-ASA is the first-line drug and widely used in clinical treatment of IBD. Remarkably, CD-LFS-01 exerts superior therapeutic efficacy to 5-ASA for experimental colitis. In particular, oral treatment of CD-LFS-01 (80 mg/kg/day) significantly restored the colon length and DAI score as compared to 5-ASA treatment group (80 mg/kg/day). More impressively, we observed that CD-LFS-01almost recovered the DSS-induced destruction of the colon mucosal layer (**Figure 7E**). In contrast, we still observed the events of epithelial cell loss and reduction of the density of the tubular glands in 5-ASA treatment group.

### Oral Treatment of LFS-01 Protects against TNBS-Induced Colitis in Rats

The animals intrarectally delivered 2,4,6-trinitrobenzene sulfonic acid (TNBS) can display clinical, histopathological and immunological features as close mimics to Crohn’s disease (CD) (Gonzalez-Rey et al., 2006; Dudley et al., 2011). We wanted to test whether LFS-01 would also be effective in a TNBS-induced rat model (Supplementary Figure S9). Strikingly, oral treatment of LFS-01 (80 mg/kg) to TNBS group of rats significantly recovered the lost body weight and reduced the morality from 80 to 30% (Supplementary Figures S9a,b). Moreover, LFS-01 oral treatment can restore the DAI score, macroscopic score, histological score and MPO activity, which are severely increased in TNBS-induced group (Supplementary Figures S9c,d,f,g). Notably, LFS-01 oral treatment clearly ameliorated the inflammatory activities and destruction of the colon mucosal layer as evidenced by the histopathological analysis (Supplementary Figure S9e). Lastly, the rats treated with LFS-01 exhibited much less incidence of diarrhea as compared to the TNBS-induced group (data not shown).

### LFS-01 Is Non-toxic to Animals

**FIGURE 8:**
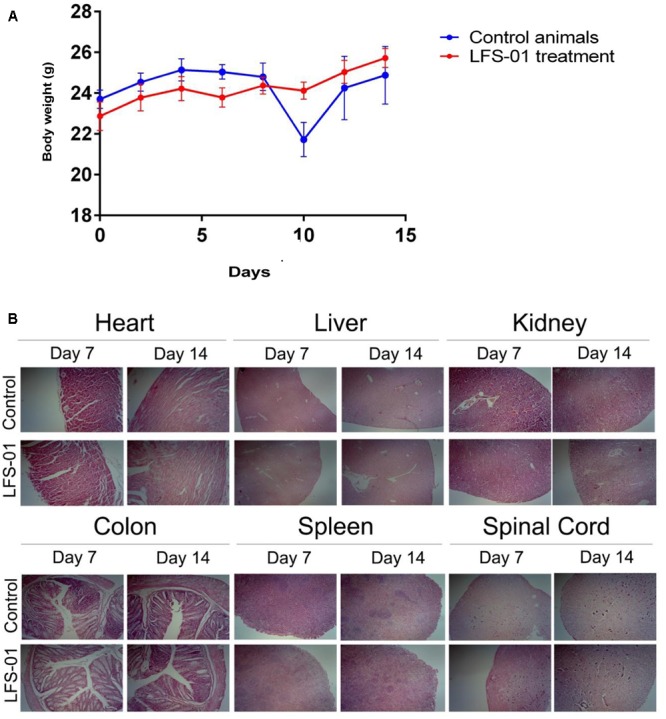
Sulforaphene does not show apparent toxicity to mice. **(A)** The *y*-axis depicts mean ± SEM body weight of animals (female and male mice) studied in the acute dosing scheme (500 mg/kg/24 h). **(B)** H&E staining for the indicated tissues in animals treated with vehicle and LFS-01(dose in 24 h: 500 mg/kg) after 7 and 14 days, respectively.

To determine its suitability as a potential IBD therapeutics in clinical practice, we examined the acute toxic effects of LFS-01 in mice. Ten BALB/c mice were exposed to oral administration of LFS-01 at a dose of 500 mg/kg over the course of 1 day whereas another 10 mice were exposed to vehicle only (70% Ethanol, *n* = 10). The drug-treated animals did not show lethargy, weight loss (**Figure 8A**) or other physical indications of sickness. The mice were sacrificed after the 14-day washout period to assess delayed toxicity. Heart, liver, kidney, colon, spleen, and spinal cord tissues were examined. No toxic effects or other signs of sickness, including weight loss or tissue damage (either macroscopic or microscopic) were observed (**Figure 8B**). These studies implicate that LFS-01 may hold promises in clinical treatment of IBD.

## Discussion

The disturbance of gut microbiota-host symbiosis is mainly responsible for the pathogenesis of IBD. In this respect, for the first time, our work reported that naturally derived sulforaphene (LFS-01) can regulate the skewed gut microbiota and enhance the production of IL-17^+^γδT cells with the expansion of *Lactobacillus*, resulting in beneficial effects on DSS-induced colitis model of mice. In particular, we identified that LFS-01 can selectively suppress the growth of harmful enteric pathogens such as *Escherichia–Shigella, Helicobacter*, and *Proteus mirabilis*. Interestingly, we demonstrated that LFS-01 barely inhibits the protective *Lactobacillus* population. The selective inhibition of enteric pathogens can be accounted for the expansion of *Lactobacillus* and *Lachnospiraceae* population after LFS-01 treatment due to the possible antagonistic relationship between commensal bacterial groups (Ramanan et al., 2016).

The concept that beneficial microflora may have evolved mechanisms to ameliorate intestinal inflammation and experimental colitis has been well accepted (Sheil et al., 2007; Sartor, 2008). For instance, the overcolonization of lactobacilli, especially *Lactobacillus murinus*, accounts for the resistance to DSS-induced colitis of animal models (Tang et al., 2015). Moreover, IBD patients were found to have a decreased population of *Lactobacillus* compared to healthy controls. Analogously, in our study, we found a decreased proportion of *Lactobacillus* in DSS-induced mice while upon LFS-01 treatment, the *Lactobacillus* population was significantly increased accompanied by the increase of IL-17^+^γδT cells which can suppress colitis. Importantly, we verified that *Lactobacillus* can directly stimulate the growth of γδT cells upon preincubation. Therefore, our work may help to explain the protective mechanisms of LFS-01 during intestinal damage. Moreover, our work endorses the role of *Lactobacillus* as a beneficial bacterial against various human disorders (Buffington et al., 2016). On the other hand, the deleterious roles of certain bacterial and fungi toward the intestinal damage and development of IBD have been proposed. For example, the clade of *Enterobacteriaceae*, particularly *Escherichia–Shigella*, has been found to be significantly increased in IBD patients and closely associated with intestinal inflammation (Mylonaki et al., 2005). The genera *Escherichia–Shigella* were also found to be particularly highly enriched in ileal CD (iCD) patients above the general abundance of CD patients (Morgan et al., 2012). Moreover, two *Enterobacteriaceae* species, *Klebsiella pneumoniae* and *Proteus mirabilis* were found to elicit colitis and contribute to the disease pathogenesis of model mice (Garrett et al., 2010). In accordance with these studies, we demonstrated that the abundance of *Escherichia–Shigella, K. pneumoniae*, and *P. mirabilis* were significantly elevated in DSS-treated mice whereas the bloom of these harmful strains was suppressed in the gut of model mice upon LFS-01 oral administration.

Our work has identified a specific fungal microbiota dysbiosis in colitis mice with shifts in composition involving the two dominant fungi phyla *Ascomycota* and *Basidiomycota* and numerous fungi genus such as *Malassezia, Fusarium, Rhodotorula, Trichosporon*, and *Cryptococcus*. In the past, very little is known about the potential impact of these fungi genus on intestinal inflammation. For instance, *Malassezia* is a genus naturally found on the skin surface of mammalians and associated with numerous skin diseases (Saunders et al., 2012). However, a few species of *Malassezia* genus which secret potent allergens have been frequently found in the human gut microbiota, suggesting their possible roles during inflammation process (Richard et al., 2015). Moreover, *Fusarium* is a genus naturally distributed in soil as well as plants and some species of *Fusarium* can secret mycotoxins that may contribute to the gut inflammation once enter the food chain (Grenier and Applegate, 2013). Our results revealed that both fungi genus of *Malassezia* and *Fusarium* are promoted in colitis model, supporting their putative roles during intestinal damage. Despite no prior reports available for the influence of fungi genus including *Rhodotorula, Trichosporon*, and *Cryptococcus* on intestinal inflammation, their potential roles during intestinal damage are emerging in our study and warrant further investigation. Remarkably, LFS-01 oral administration reversed the over-colonization of these fungi genus in DSS-treated mice. Hence, our data reinforce the concept that fungi are important co-factors in gut microbiological homeostasis and the distortion of fungi microbiota may aggravate the colitis.

Recent studies have implicated that commensal microbiota can directly regulate the lymphocyte populations of γδT cells (Ismail et al., 2009), which play critical roles in the protection of epithelial barriers and defense against pathogens following mucosal injury. Importantly, we demonstrated that LFS-01 administration significantly promoted the specific population of IL-17^+^γδT cells in response to alerted microbiota composition and enhanced the production of IL-17A in the cLP of model mice. Our data concurs with prior published work (Lee et al., 2015) that IL-17^+^γδT cells in colon mucosa are crucial for barrier protection during DSS injury. We showed that LFS-01 oral administration restored the excessive epithelial permeability within the colon of DSS-treated mice. This is in accordance with the fiasco of two recent clinical trials with secukinumab (Hueber et al., 2012) targeting IL-17A and brodalumab (Gaffen et al., 2014) targeting IL-17RA, both of which show no efficacy or even higher rates of adverse events in IBD patients. Further, our results established that LFS-01 treatment promotes the production of γδT cells mediated through TLR2 pathway. This is in line with prior reports (Akira et al., 2006; Round et al., 2011) that TLR2 pathway is critical for recognizing microbial products and activating innate immune system in responses to altered microbiota. We revealed that the expansion of bacterial *Lactobacillus* is mainly responsible for the promotion of γδT cells upon LFS-01 treatment.

Our data demonstrated that LFS-01 is effective in DSS-induced colitis model of mice as well as TNBS-induced colitis model of rats, two mostly widely used chemically induced rodent models to mimic UC and CD, respectively. Moreover, the pathological characteristics of IBD dictates that the onset of the disease may translate into longstanding disease activity and thereby require life-long medication. Hence, special attention is warranted for the IBD drug toxicity. For this respect, we assessed the acute toxicity of LFS-01 in animals and our results suggest that LFS-01 may hold potentials as IBD therapy in clinical practice without apparent toxicity. Further, to enhance the oral bioavailability, we prepared the CD-encapsulated LFS-01 (CD-LFS-01) as stable and water-soluble powder and our results confirmed its efficacy in colitis model of mice. Importantly, CD-LFS-01 is superior to 5-ASA for recovering intestinal injury at the same dose. To our knowledge, our work is the first to harness the microbiota-host crosstalk toward therapeutic ends for treating IBD. Our results strongly implicate that LFS-01 oral administration reverses the course of IBD through restoring gut microbiota-host symbiosis.

Sulforaphene is the major chemical ingredient derived from *R. sativus*, a medicinal herb documented in the books of Traditional Chinese Medicine to treat symptoms such as food stagnation and cough. However, to the best of our knowledge, the use of *R. sativus* for the treatment of IBD has not yet been documented. Nevertheless, it is conceivable that *R. sativus* or its crude extracts may also have therapeutic effects on IBD. The advantage of using LFS-01 or its synthetic analogs over *R. sativus* in clinical practice is that only a small dose is required for the IBD patients. Another merit of LFS-01 as compared to the first-line IBD drugs is that LFS-01 may have better safety profile in human because *R. sativus* has been used for over a 1000 years in China. Presently, we are developing synthetic analogs for LFS-01 with better efficacy for IBD treatment and will report the results in due course.

## Conclusion

In summary, our work shed new light on poorly understood interplay of microbiota-host which has a significant impact on intestinal injury. We demonstrated that oral treatment of LFS-01 holds great promise as an effective remedy for IBD with excellent safety profile. In the meantime, we are investigating the structural analogs of LFS-01 for IBD treatment with better efficacy yet less dosage. Our work provides a transnationally relevant implication for developing LFS-01-based therapies as next-generation strategies for IBD prevention and treatment.

## Author Contributions

MLi, YT, and YiL performed the colitis models, animal studies and acute toxicity studies. JG, HL, and MLiu performed *in vitro* enzymatic assay studies. MeL, YuL, and XL performed LFS-01 formulation and encapsulation studies. KQ performed *in vitro* radial diffusion assay studies. SW performed bioinformatics analysis and modeling studies. XZ and JW performed virtual screening studies and participated in figure preparation. LM and MLi performed the 16S rDNA and ITS data analysis and participated in figure preparation. EW, JY, and JW participated in critical revision of the manuscript. YY led the project, oversaw the manuscript preparation and wrote the manuscript.

## Conflict of Interest Statement

LM serves as Chief Computing Officer for Driving Force Therapeutics (DFT); YY is a scientific Co-founder of DFT; JW and MLi serve as scientific advisors to DFT. The other authors declare that the research was conducted in the absence of any commercial or financial relationships that could be construed as a potential conflict of interest.
