# Supplementary material for: Traditional Herbal Medicine-Derived Sulforaphene LFS-01 Reverses Colitis in Mice by Selectively Altering the Gut Microbiota and Promoting Intestinal Gamma-Delta T Cells
Source: Front Pharmacol. 2018 Jan 9;8:959. doi: 10.3389/fphar.2017.00959 (PMC5767259; doi:10.3389/fphar.2017.00959)
Supplement: Supplementary file 2 [file Data_Sheet_1.docx]

**Supplementary Information**

**Fig. S1. The specific bacterial groups that were significantly regulated by LFS-01 treatment.** The abundance of different microbial groups at family, genus and species level between groups was tested by means of MetaStat method through the 16srDNA sequencing experiments. The resulted *p* values were further adjusted to obtain q values. * *q* value < 0.05; ** *q* value < 0.01.

**Fig. S2. The harmful bacterial strains and protective bacterial strains that were significantly modulated by LFS-01 treatment.** Upper panel**,** the average percentage of abundance for two harmful bacterial groups through the 16srDNA sequencing experiments; lower panel, the average percentage of abundance for two beneficial bacterial groups through the 16srDNA sequencing experiments.

**Fig. S3. Heatmaps indicating the abundance of bacteria microbiota at different levels in each animal group.** All presented results are statistically significant(p<0.05) as evaluated by the Wilcoxon test.

**Fig. S4. LFS-01 treatment modulates intestinal fungal microbial composition in model mice. (a)**. Venn diagram of shared and independent fungal OTUs in different experimental groups (n=7); **(b)**. Principal Coordinate Analysis (PCoA) based on weighted Unifrac distances among different samples. PC1 and PC2 account for 81.20% of the variation; **(c)**. Heatmap of the abundance of the dominant genera in different groups. The relative abundances of each genera were normalized and the Z-value was presented and depicted by the color intensity; **(d-e)**. Microbial composition that are different among different experiment groups were identified using MetaStat method. * adjusted p value < 0.05; ** adjusted p value < 0.01.

**Fig. S5. Levels of cytokines in colon tissue of mice in different experimental groups.** The protein levels of the pro- and anti-inflammatory cytokines in colon tissue of mice were measured by ELISA (USCN). * p< 0.05, ** p <0.01, *** p < 0.001.

**Fig. S6. LFS-01 treatment doesn’t impact the dectin-1 expression of γδT cells.** Cells in colonic LNs isolated from mice (n=7) were analyzed by flow cytometry. **(a)** CD4^-^TCR-γδ^+^cells were gated and the expression of the Dectin-1 was measured. **(b)** The presence of TCR-γδ^+^ cells in CD4^-^TCR-γδ^+^cells. n.s. no significant differences were detected.

**Fig. S7. The ^1^H-NMR spectra data for water-soluble formulation CD-LFS-01. (a)** ^1^HNMR spectra data for α-Cyclodextrin; **(b)** ^1^HNMR spectra data for LFS-01; **(c)** ^1^HNMR spectra data for CD-LFS-01 encapsulated complex.

**Fig. S8. The IR spectra for CD-LFS-01**.

**Fig. S9. LFS-01 treatment ameliorates TNBS-induced colitis in rats.** Colitis was induced by intra-colonic treatment of TNBS (100 mg/kg per rat) in 50% ethanol. Rats were treated with LFS-01 (80 mg/kg) 12 h after TNBS injection. Rats treated with 50% ethanol were used as controls. Clinical severity was assessed by **(a)** survival, **(b)** original weight **(c)** and DAI score; The macroscopic-damage score **(d)** was determined at 3 days post TNBS administration; **(e-f)** Histopathological analysis was performed in H&E sections of rat colons at day 3 (Magnification, 200). **(g)** MPO enzyme activity was measured in colonic tissues.

**
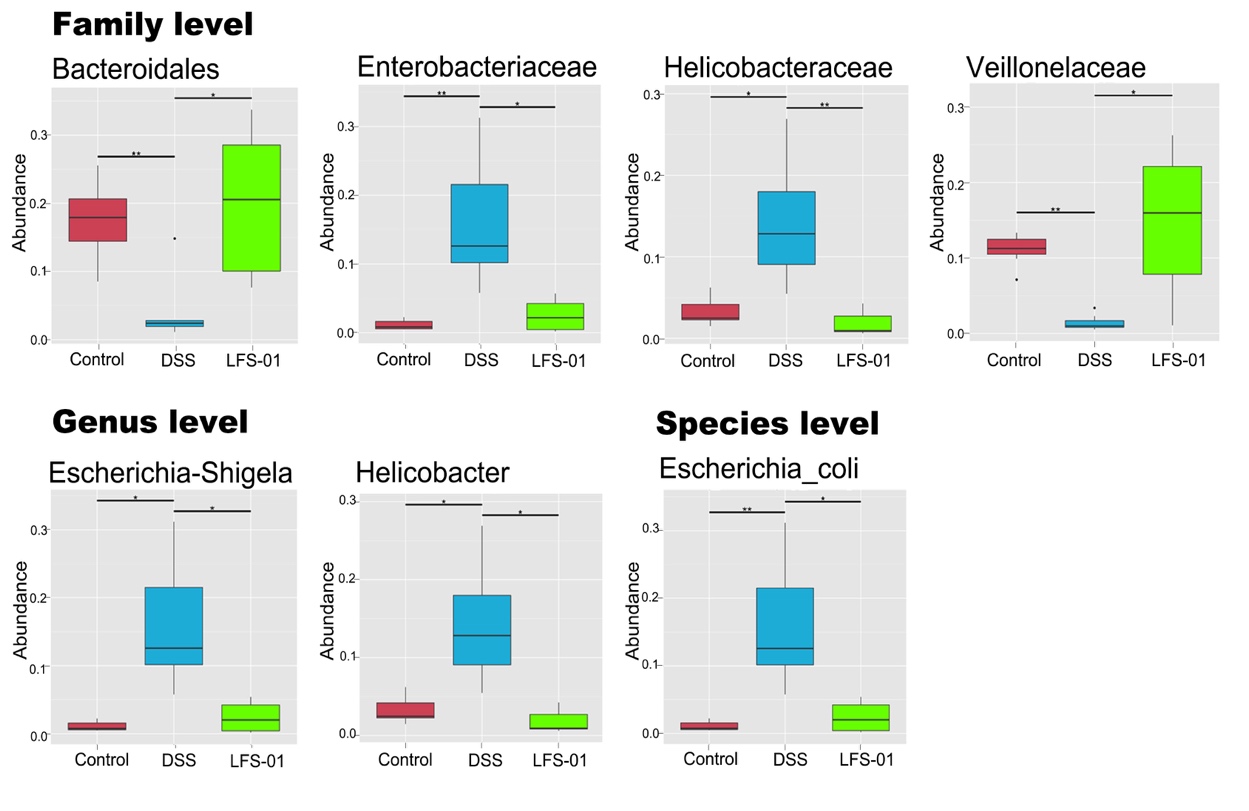
**

**Fig. S1.**


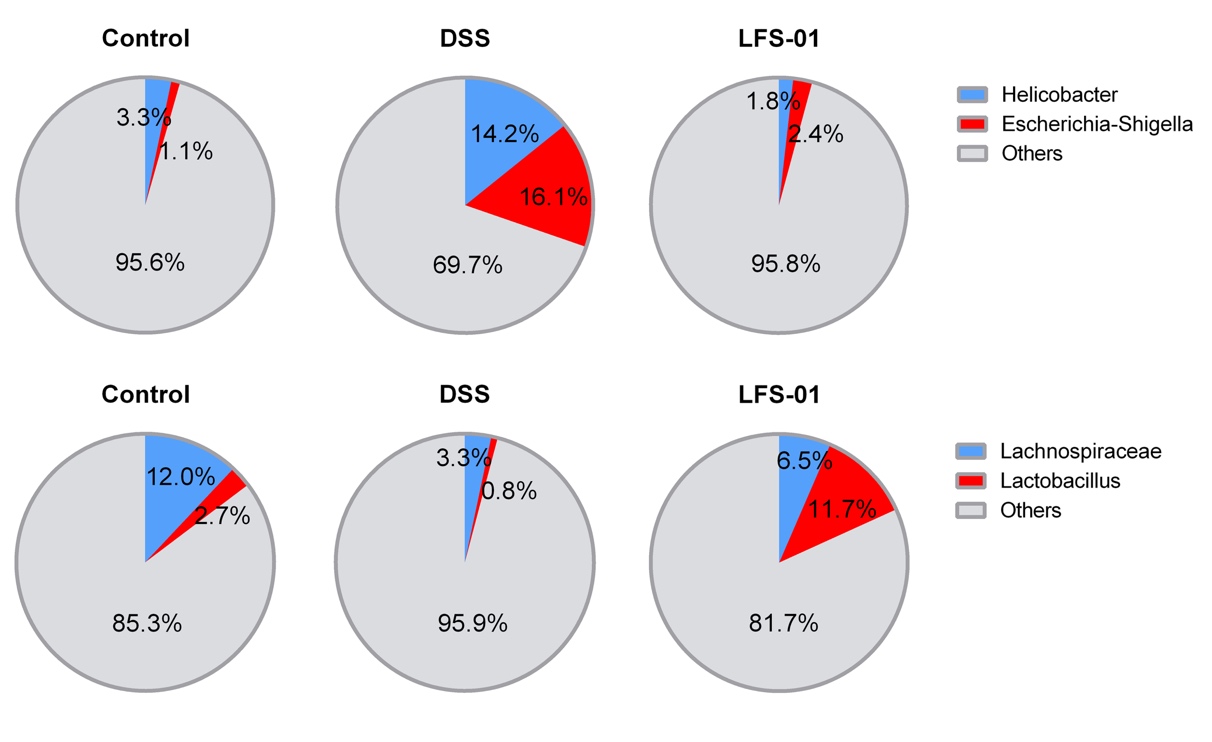


**Fig. S2.**

**
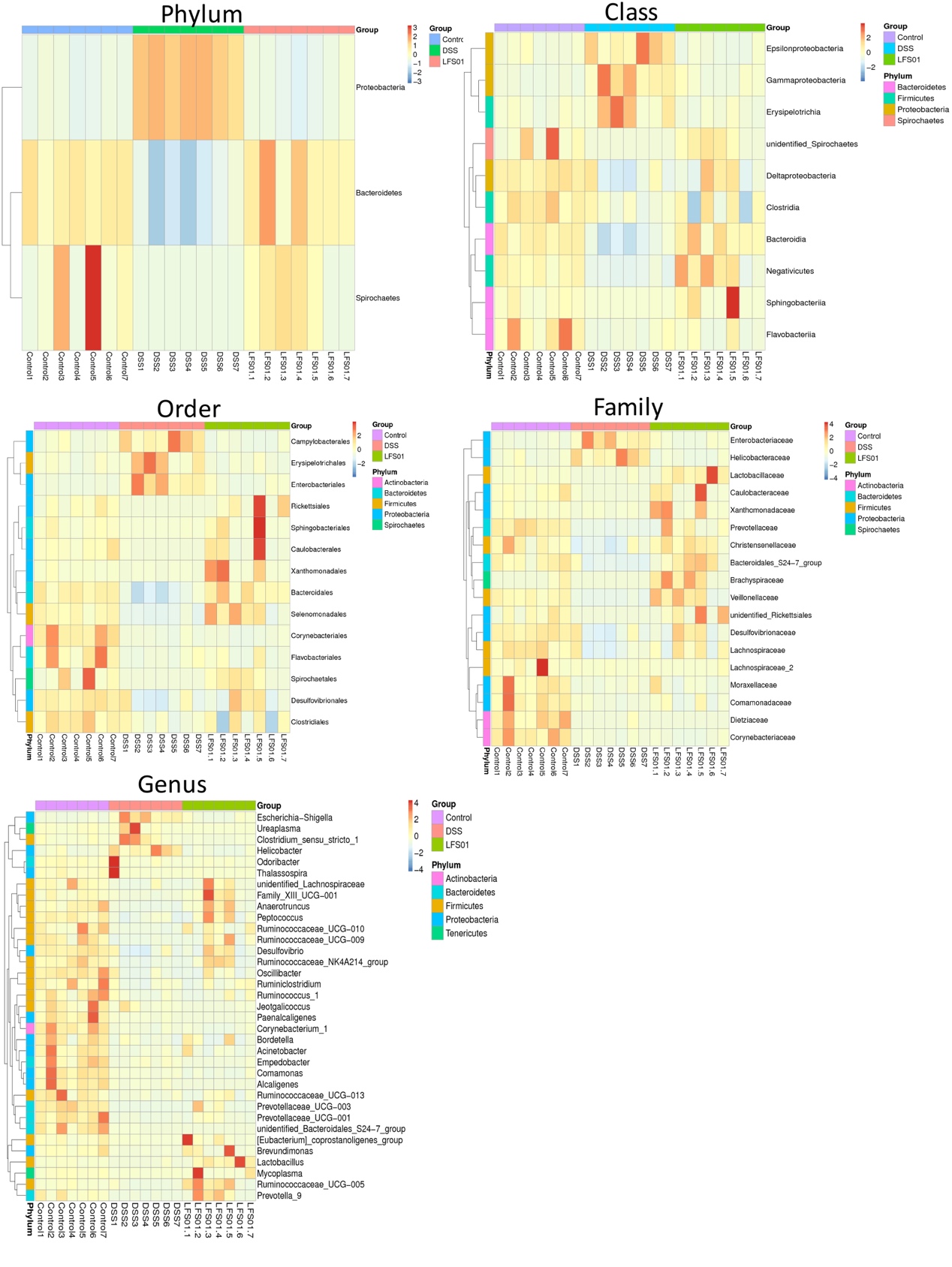
**

**Fig. S3.**


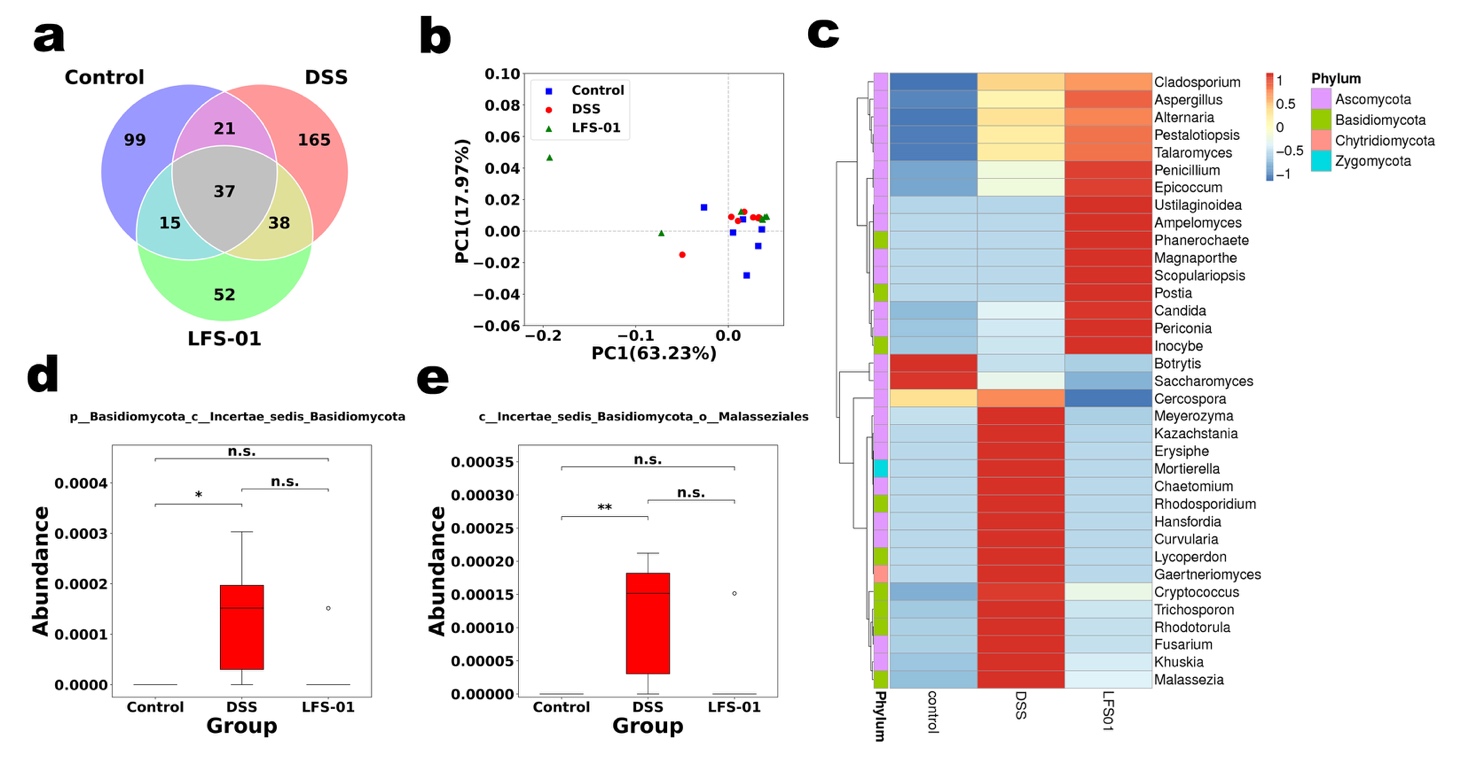


**Fig. S4.**


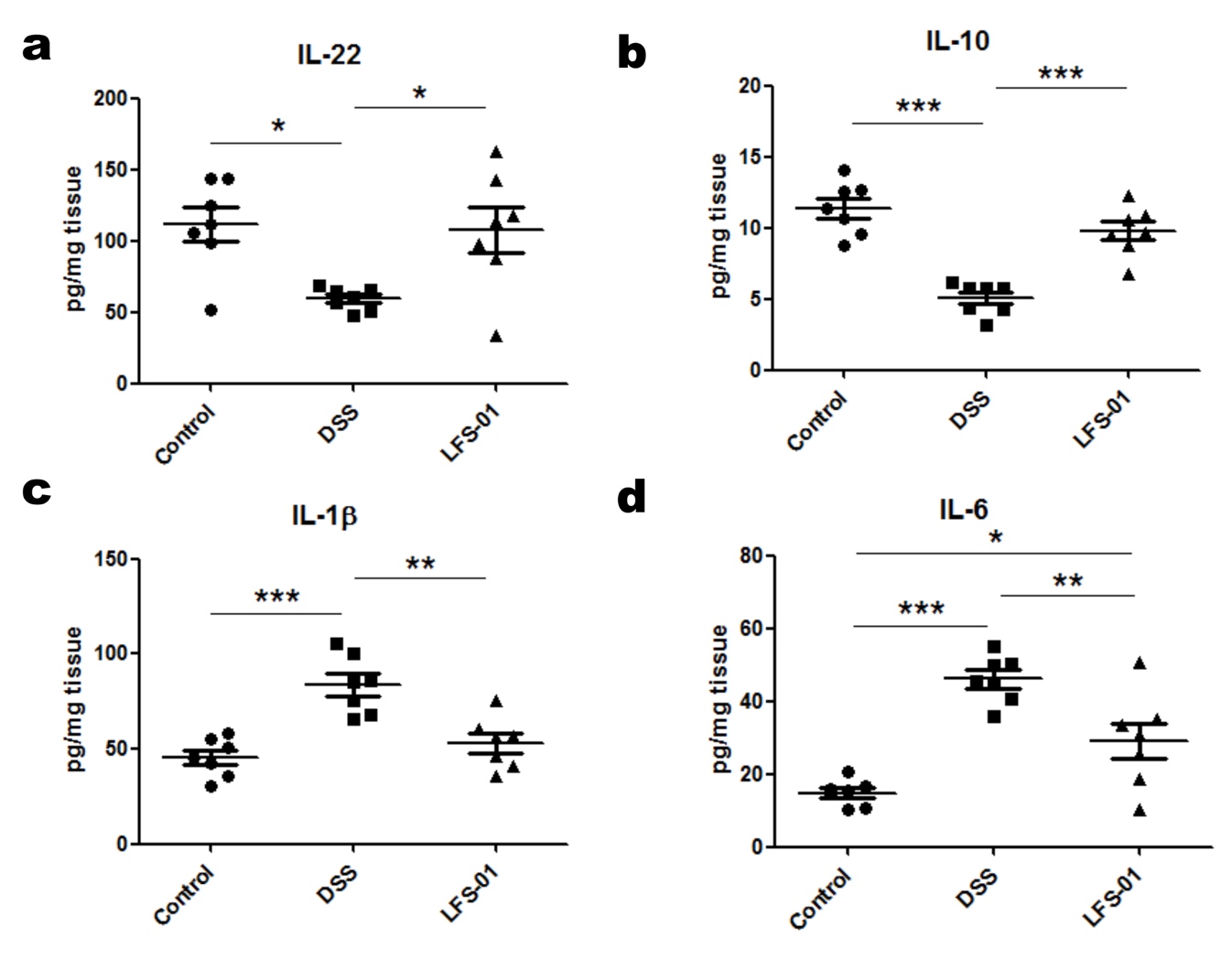


**Fig. S5.**

**
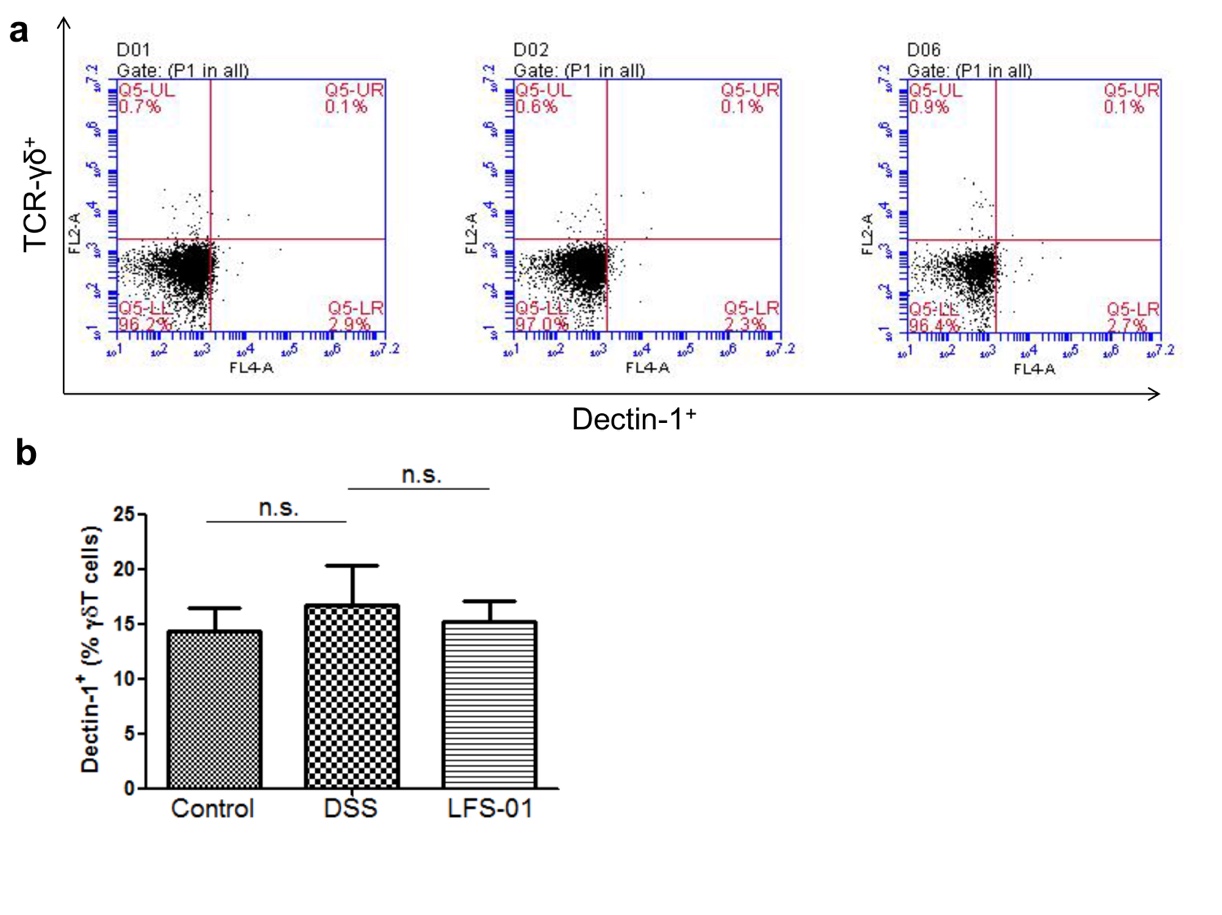
**

**Fig. S6.**

**
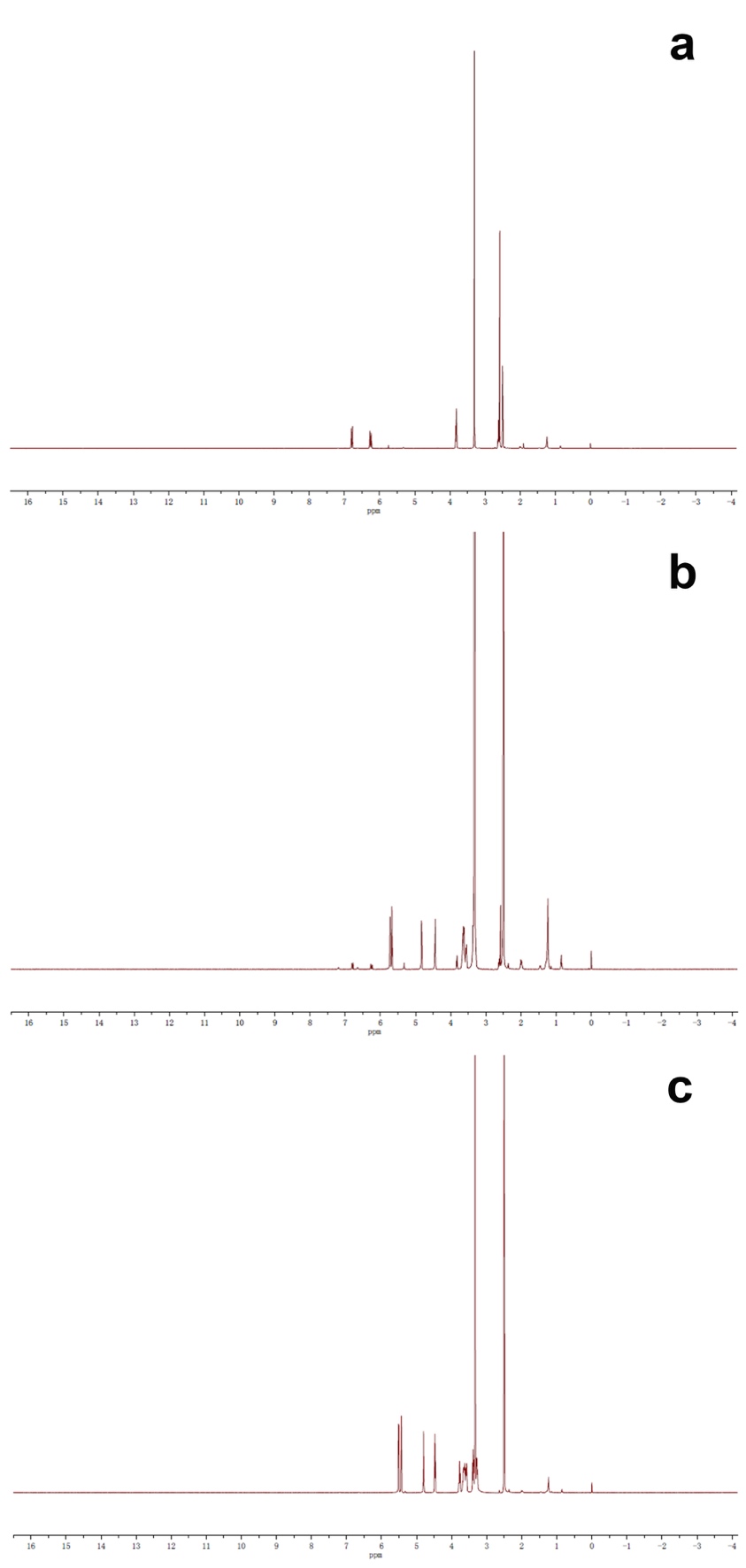
**

**Fig. S7.**

**

**

**Fig. S8.**

**
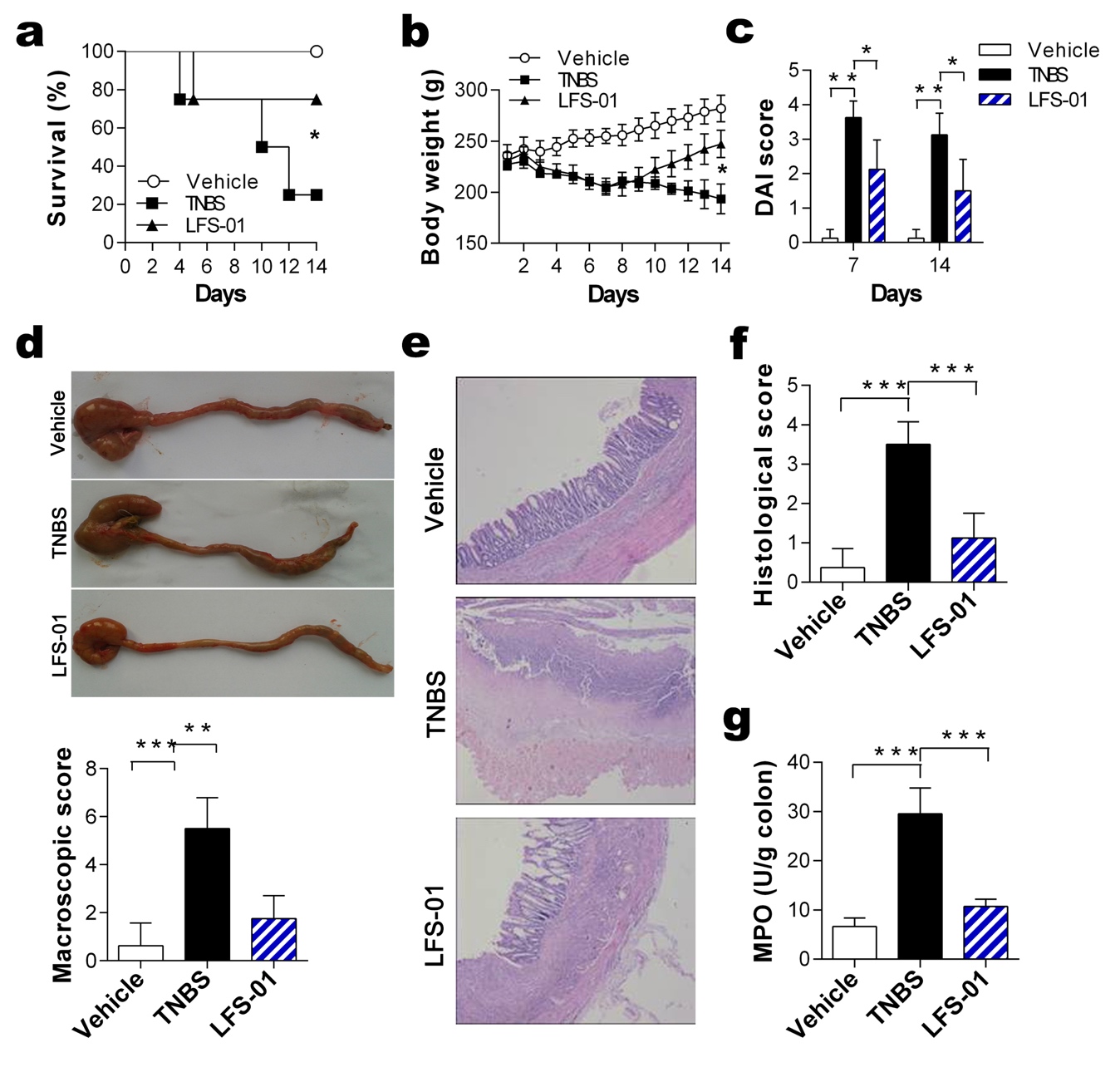
**

**Fig. S9.**
